# Hypothyroidism is a causal determinant of age-related cataract risk in European population: a Mendelian randomization study

**DOI:** 10.3389/fendo.2024.1254793

**Published:** 2024-02-05

**Authors:** Shu Liu, Qi Sun, Qingwei Gu, Yujie Bao, Wei Wang, Xiaodong Qin, Xinran Yuan

**Affiliations:** ^1^ Department of Rheumatology and Immunology, Nanjing Drum Tower Hospital, Affiliated Hospital of Medical School, Nanjing University, Nanjing, China; ^2^ Department of Endocrinology, Nanjing First Hospital, Nanjing Medical University, Nanjing, China; ^3^ Department of Orthopedic Surgery, Nanjing Drum Tower Hospital, Affiliated Hospital of Medical School, Nanjing University, Nanjing, China

**Keywords:** thyroid, hypothyroidism, age-related cataract, Mendelian randomization, genome-wide association study

## Abstract

**Objective:**

To determine whether there is a causal relationship between thyroid dysfunction and the risk of age-related cataract (ARC) in the European population.

**Design:**

A two-sample Mendelian randomization (MR) study.

**Methods:**

Hypothyroidism, hyperthyroidism, free thyroxine (fT4), and thyrotropin (TSH) were selected as exposures. The single nucleotide polymorphisms (SNP) of hypothyroidism and hyperthyroidism were obtained from the genome-wide association studies (GWAS) of the IEU database, including 337,159 subjects. Data for fT4 and TSH (72,167 subjects) were extracted from the ThyroidOmics Consortium. ARC was used as the outcome. The SNPs associated with ARC were selected from a GWAS of 216,362 individuals in the FinnGen database. The main method used was the inverse variance-weighted method, together with four complementary methods. Sensitivity analyses were performed using Cochran’s Q test, MR-PRESSO, MR-Egger regression and leave-one-out test. MR pleiotropy was used to test for pleiotropy. MR Steiger test was used to test for the directionality.

**Results:**

Two-sample MR analysis revealed a positive association between genetically predicted hypothyroidism and risk of ARC (OR = 2.501, 95% CI: 1.325-4.720; *P* = 0.004). Hyperthyroidism, circulating fT4 and TSH levels did not have a significant causal effect on ARC (*P* > 0.05). The results were robust and reliable, and no horizontal pleiotropy was found after sensitivity analyses. In the MR Steiger test, we found no reverse causal effects of hypothyroidism on the ARC (*P <*0.001).

**Conclusions:**

Our study provides strong evidence that hypothyroidism is a causal determinant of ARC risk.

## 1 Introduction

Age-related cataract (ARC) also known as senile cataract, is the most common form of cataract and a leading cause of vision impairment worldwide (1). It is characterized by the progressive formation of an opaque lens that eventually leads to loss of vision and eventual blindness (2, 3). It typically affects people over the age of 60, but can also affect younger people (1). Sight can be restored through cataract removal surgery, in which the clouded lens is removed and an artificial lens is implanted. Although the success rate of cataract surgery is high, there are risks associated with cataract surgery, including lens fragments, vitreous detachment or traction, retinal detachment, intraocular inflammation or hemorrhage, corneal edema, infection, and increased intraocular pressure (4–6). In addition, the cost of cataract surgery to remove ARC remains a significant burden for many families. Statistics show that nearly 2 million cataract surgeries are performed every year in the United States at a cost of approximately $60 billion, and that delaying the onset of ARC by 10 years can reduce the number of people requiring surgery by 45 percent (6, 7). Therefore, it is essential to identify potential risk factors for delaying or preventing ARC in order to improve patients’ quality of life and reduce the immense social and economic burden.

As a type of bioregulatory hormone, thyroid hormone (TH) plays crucial roles in human growth and development, metabolism, cardiovascular function, and temperature regulation (8). It circulates in the balance between the biologically active free form and the protein-bound form, the production and release of which is primarily regulated by the hypothalamus and pituitary gland (9). TH may play a crucial role in the pathogenesis of eye diseases. TH signaling has been shown to play a critical role in the regulation of retinal development and is a key regulator of S- and M-cone opsins (10). Additionally, the absence of TH during embryonic and early postnatal development can also affect the formation of the eyeball and the proliferation of all retinal cells (11, 12). Even when an individual’s thyroid function is within the normal range, higher FT4 levels are associated with an increased risk of age-related macular degeneration (AMD), according to recent research (13). However, the function of TH in ARC has been studied little. A case-control study of risk factors for ARC showed that moderate cortical opacities were associated with higher levels of thyroid hormone use (14). In another study, it was also found that metabolic cataracts could be treated with TH supplements (15). Although both studies attempted to control for potential confounding variables, the causal relationship between thyroid dysfunction and ARC remains unclear.

Mendelian Randomization (MR) is a statistical method in epidemiology that uses genetic variation as instrumental variables (IVs) to infer causal relationships between exposures and outcomes (16). During gamete formation, alleles are segregated and are randomly distributed to offspring according to Mendel’s law of independent assortment (17). This suggests that modifying exposure through genetic variation will have an identical effect on the outcome. A number of conditions, such as atrial fibrillation, renal function, anemia, sex hormones and function, and major depressive disorder and its subtypes, have been predicted to be affected by thyroid dysfunction (18–21). There are currently no Mendelian Randomization (MR) studies investigating the causal relationship between thyroid dysfunction, specifically hypothyroidism, and the risk of ARC. To determine the causal relationship between thyroid dysfunction (including hypothyroidism, hyperthyroidism, circulating fT4 and TSH levels) and ARC risk, we performed an MR study using Single Nucleotide Polymorphisms (SNPs) as IVs.

## 2 Materials and methods

### 2.1 Study design and data sources

As the data for our study were obtained from publicly available databases, no consent was required from subjects. This study followed the Strengthening the Reporting of Observational Studies in Epidemiology–Mendelian Randomization reporting guidelines (**Supplementary Table S1**, available on the *Jama Network* at https://jamanetwork.com/journals/jama/fullarticle/2785494) (22).

We conducted a two-sample MR study to investigate the causal relationship between thyroid dysfunction (including hypothyroidism, hyperthyroidism, circulating fT4 and TSH levels) and ARC. Hypothyroidism and hyperthyroidism were derived from a large genome-wide association study (GWAS) conducted by the Neale laboratory in 2017, accessible via the IEU database (https://gwas.mrcieu.ac.uk/). The code for hypothyroidism was UK-Biobank (UKB)-77, which is defined as self-reported hypothyroidism with non-cancer disease, and the code for hyperthyroidism was UKB-76, which is defined as self-reported hyperthyroidism/thyrotoxicosis. There were 16,376 samples and 320,783 controls for hypothyroidism and 2,547 samples and 334,612 controls for hyperthyroidism. The GWAS data for fT4 and TSH were obtained from the ThyroidOmics Consortium database, which contained 72,167 samples. The ThyroidOmics Consortium is an international consortium investigating the determinants and influences of thyroid disease and function and has assembled the largest and most recent GWAS data for normal-range circulating fT4 and TSH levels of European ancestry (23).

ARC was derived from summary-level statistics provided by the FinnGen consortium in 2021, which included 216,362 samples (26,758 cases and 189,604 controls) and 16,380,461 SNPs. The complete GWAS of ARC is available to the public through the FinnGen research project (https://www.finngen.fi/en), which aims to evaluate genotype-phenotype correlations in order to identify novel therapeutic targets. The diagnosis of ARC was based on International Classification of Diseases codes, including ICD-9 366.1 and ICD-10 H25, and was defined by a visual acuity of 0.7, lens opacity, and the absence of other eye diseases causing visual impairment.

All of the above GWAS studies were restricted to people of European descent.

### 2.2 Assumptions of MR study

**Figure 1:**
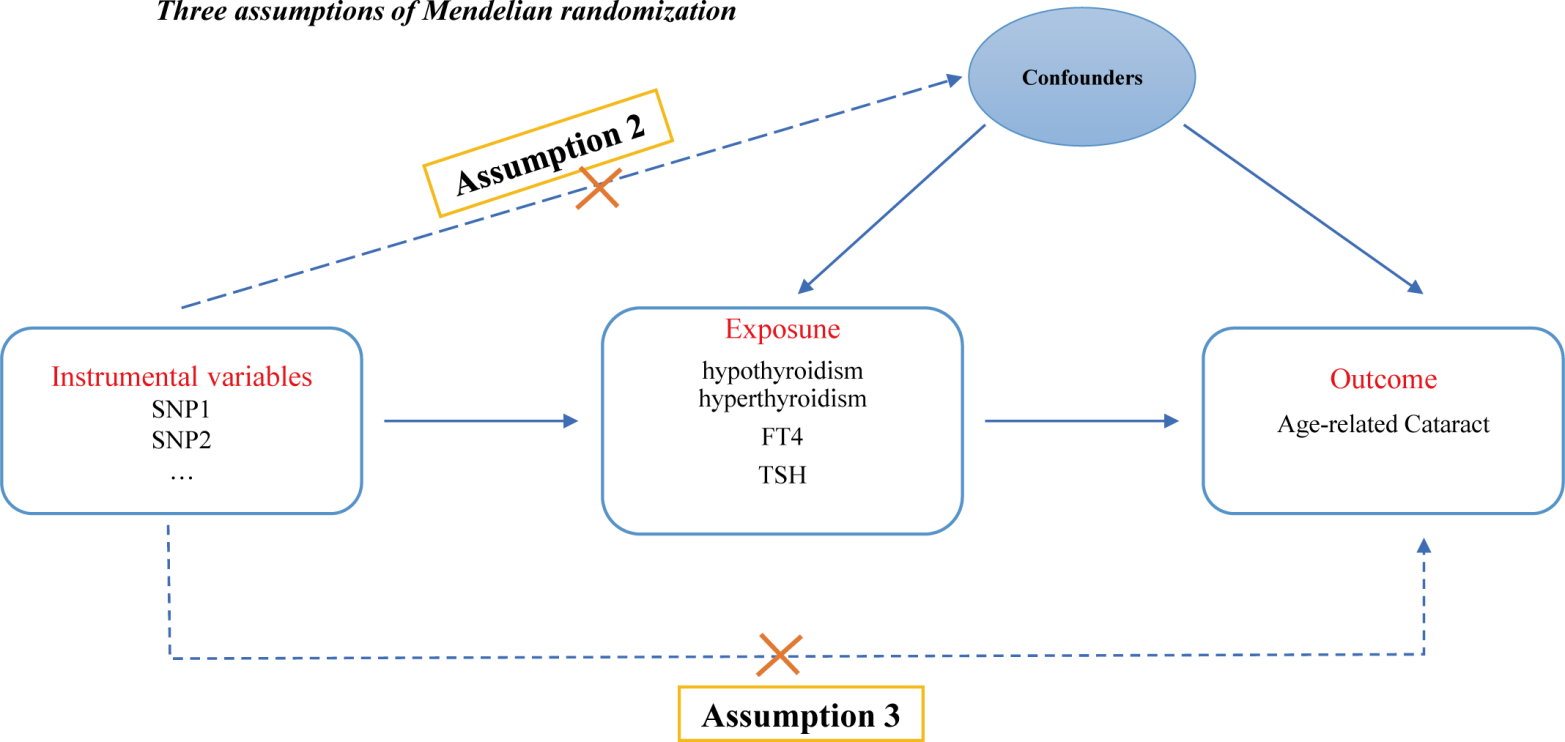
Directed acyclic graph of the MR framework examining the cause-and-effect relationship between thyroid dysfunction and the risk of ARC. Assumption 1: Instrumental variables should be robustly associated with exposure. Assumption 2: Instrumental variables should not be associated with any confounders. Assumption 3: Instrumental variables should not be associated with ARC except through exposure.

This study strictly adhered to the three key assumptions of MR analysis: (1) the genetic IVs must be closely associated with thyroid dysfunction (including hypothyroidism, hyperthyroidism, circulating fT4 and TSH levels) (the relevance assumption); (2) the genetic instruments used should not be associated with potential confounders of ARC (the independence assumption); and (3) genetic variation should only affect the risk of ARC through thyroid dysfunction (the exclusivity assumption) (24). **Figure 1** illustrates the design of the MR analysis and the underlying assumptions.

### 2.3 Instrumental variables selection

IVs were selected based on the three hypotheses presented in **Figure 1**. First, we searched for IVs that showed a significant correlation with the exposure (*P* < 5×10^-8^). To eliminate linkage disequilibrium between each SNP, we set the distance to 10,000 kb and the LD r^2^ to 0.001. We then standardised the SNPs for the exposure and outcome and removed SNPs that were incompatible or duplicated. The F statistic was then calculated for each SNP (F=β^2^_exposure_/SE^2^_exposure_), and SNPs with an F value of less than 10 were eliminated (25). Furthermore, we used the Phenoscanner ((http://www.phenoscanner.medschl.cam.ac.uk/), with the threshold set at *P* < 5 × 10^-8^ and the LD r^2^ of 0.001, to exclude confounding SNPs associated with outcome. SNPs associated with ocular trauma, age, cortisol use, ultraviolet radiation, and calcium metabolism disorders are considered confounding SNPs.

### 2.4 Statistical analyses

We primarily used the Inverse Variance Weighted (IVW) method to assess the causal relationship between thyroid dysfunction and ARC risk. In addition, MR-Egger and weighted median were selected as complementary methods. The odds ratio (OR) as well as 95% confidence interval (CI) of ARC correspond to the genetically determined increase in thyroid dysfunction. The IVW method provides the most accurate estimate, but it is susceptible to invalid IVs and pleiotropic effects (26). Therefore, we performed additional sensitivity analyses based on the MR-Egger, MR-Pleiotropy Residual Sum, and Outlier (MR-PRESSO) methods to explore and correct for potential pleiotropy, with *P*-values greater than 0.05 indicating the absence of horizontal pleiotropy (27). We used Cochrane’s Q test to assess for heterogeneity, and if heterogeneity was present, we estimated the main effect using the IVW random effects method (28). In addition, we used the MR-PRESSO analysis to identify any outliers that may have contributed to heterogeneity (29). For the MR-PRESSO analysis, we used the MR-PRESSO package with default parameters (SignifThreshold = 0.05, NbDistribution = 1000). If any outliers were identified, we removed them and re-evaluated the MR effect. The MR Steiger test was used to validate the directionality of exposure to outcome, and a *P* value of less than 0.05 was considered statistically significant (30). All two-sample MR analyses in this study were based on the TwoSampleMR package in the R software (version 4.30).

## 3 Results

### 3.1 Selection of instrumental variables

A total of eighty-three SNPs related to hypothyroidism were extracted (*P* < 5.0×10^−8^, LD r^2^ < 0.001). Two SNPs were lost in the resulting search, rs2921053 was removed as it was a palindromic SNP, and no confounding SNPs were found. Six SNPs related to hyperthyroidism (*P* < 5.0×10^−8^, LD r^2^ < 0.001) were extracted. Two SNPs were lost in the search for outcomes, and no confounding or palindromic SNPs were found. Thirty-one SNPs related to FT4 (*P* < 5.0×10^−8^, LD r^2^ < 0.001) were extracted. Ten SNPs were lost in the search for outcomes, one SNP was deleted because it was a palindromic SNP, and another SNP was excluded because it was related to mineral metabolism disorders. Sixty-one SNPs related to TSH (*P* < 5.0×10^−8^, LD r^2^ < 0.001) were extracted, and twenty-three SNPs were lost in the search for outcomes, and there were no confounding or palindromic SNPs. The MR-PRESSO global test detected no evidence of pleiotropic effects (*P* > 0.05). All SNPs used as IVs had F-statistics greater than 10 (**Supplementary Tables S2**-**S5**), indicating that the IVs were effectively implemented. Finally, after a series of quality assessments, eighty SNPs for hypothyroidism, four for hyperthyroidism, nineteen for fT4, and thirty-eight for TSH were selected as effective IVs (**Supplementary Tables S2**-**S5**).

### 3.2 Two-sample MR analysis for evaluating causal effects of thyroid dysfunction on ARC

**Figure 2:**
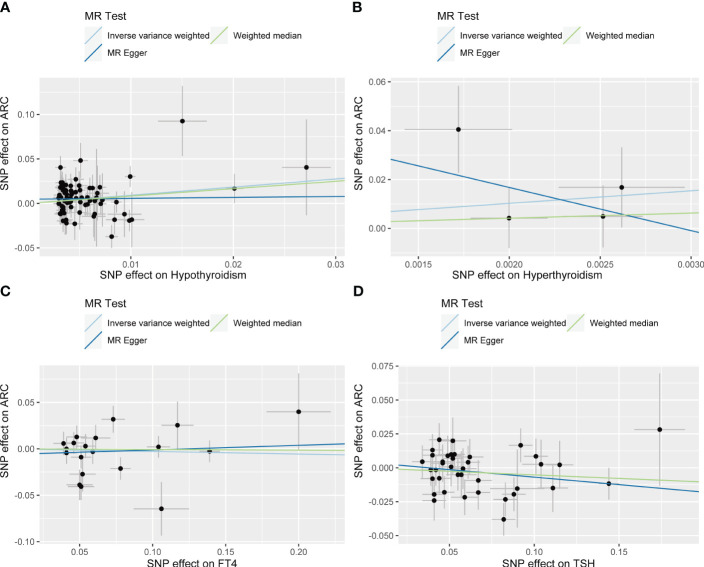
Scatter plots for Mendelian randomization (MR) analyses of the causal relationship between thyroid dysfunction and ARC. **(A)** Hypothyroidism- ARC. **(B)** Hyperthyroidism- ARC. **(C)** f T4- ARC. **(D)** TSH- ARC.

**Figure 3:**
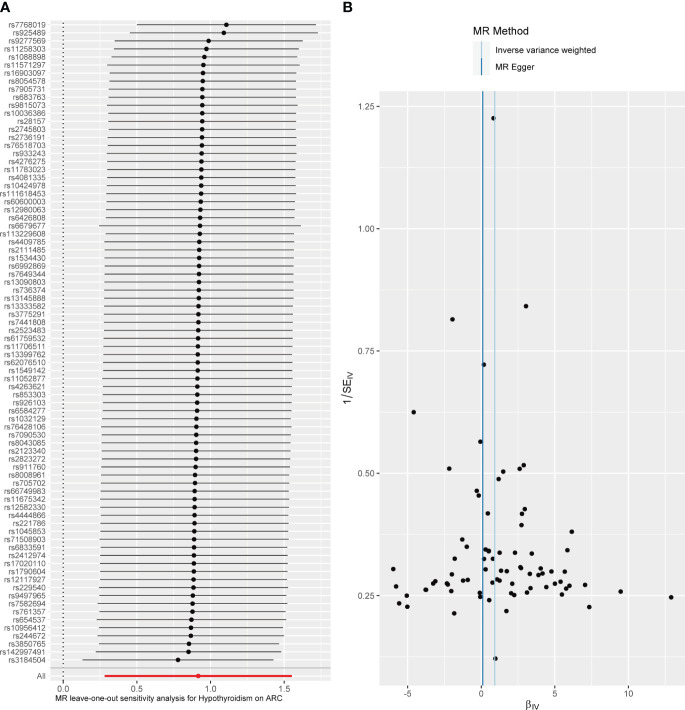
Diagnostic plot of a two-sample Mendelian randomization analysis for hypothyroidism-ARC. **(A)** Funnel plot for individual causal effect estimates. **(B)** Forest plot for leave-one-out analysis, where each point reprsents the causal effect by IVW after removing the specific SNP on the left side.

IVW showed that hypothyroidism increased the risk of ARC (OR = 2.501, 95% CI, 1.325-4.720; *P* = 0.004); However, MR Egger and weighted median found no evidence of the association between hypothyroidism and ARC (MR Egger, *P* = 0.881; weighted median, *P* = 0.115; **Table 1**; **Figure 2**). The MR-Steiger test showed that the effect of hypothyroidism on ARC was in the correct causal direction (*P*<0.001). In addition, none of the three methods above showed a statistically significant causal association between hyperthyroidism, circulating fT4 and TSH levels and the risk of ARC (**Table 2**; **Figure 3**).

**Table 1:** MR estimates from different methods of assessing the causal effect of thyroid dysfunction on ARC.

Exposure	MR methods	nSNP	Beta	OR (95% CI)	*P*-value
Hypothyroidism	IVW	80	0.916	2.501 (1.325, 4.720)	0.004
	MR-Egger	80	0.101	1.107 (0.275, 4.458)	0.886
	Weighted median	80	0.828	2.291 (0.815, 6.438)	0.115
Hyperthyroidism	IVW	4	5.130	1.690e+02 (1.536e-01, 1.859e+05)	0.151
	MR-Egger	4	-17.676	2.106e-08 (3.950e-29, 1.123e+13)	0.543
	Weighted median	4	2.087	8.063e+00 (4.745e-03, 1.370e+04)	0.565
fT4	IVW	19	-0.028	0.972 (0.865, 1.093)	0.638
	MR-Egger	19	-0.050	1.051 (0.791, 1.396)	0.735
	Weighted median	19	-0.007	0.993 (0.881, 1.119)	0.906
TSH	IVW	38	-0.052	0.949 (0.888,1.014)	0.121
	MR-Egger	38	-0.111	0.895 (0.754,1.062)	0.214
	Weighted median	38	-0.052	0.998 (0.876,1.138)	0.317

fT4, free thyroxine; TSH, thyrotropin; ARC, Age-related cataract; SNP, single nucleotide polymorphism; MR, Mendelian randomization; IVW, inverse variance weighting; All IVW P-values come from the random effects model.

**Table 2:** Sensitivity analysis of thyroid dysfunction causally linked to ARC.

Exposure	Outcome	Pleiotropy		Heterogeneity		Outlier examination by MR-PRESSO	
		Horizontal pleiotropy (Egger intercept)	Horizontal pleiotropy (*P*-value)	Heterogeneity (Q)	Heterogeneity (*P*-value)	Before correction (*P*-value)	After correction (*P*-value)
Hypothyroidism	ARC	0.005	0.209	96.645	0.086	0.005	NA
Hyperthyroidism	ARC	0.052	0.443	3.848	0.278	0.247	NA
fT4	ARC	-0.006	0.56	33.178	0.016	0.822	NA
TSH	ARC	0.004	0.475	40.661	0.312	0.130	NA

fT4, free thyroxine; TSH, thyrotropin; ARC, Age-related cataract; MR, Mendelian randomization.

### 3.3 Heterogeneity, pleiotropy, and sensitivity analysis

When hypothyroidism was the exposure, neither heterogeneity (*P* = 0.086) nor pleiotropy (*P* = 0.209) was observed. The results of the MR-PRESSO test for potential outliers were consistent with those of the IVW test (**Table 2**). The leave-one-out analysis and funnel plot further confirmed the stability of the results, as shown in **Figure 3**. We therefore considered the IVW results to be credible.

When circulating fT4 levels were considered as exposure, we observed heterogeneity (*P* = 0.016), whereas hyperthyroidism and TSH circulating levels showed no heterogeneity. If there is heterogeneity, the random-effects model in IVW should be chosen (29). Further MR-Egger regression and MR-PRESSO outlier detection analyses revealed neither pleiotropy nor outliers for hyperthyroidism, circulating fT4 and TSH levels (**Table 2**). Finally, the leave-one-out test, funnel plot, and forest plot provided additional confirmation of the results (**Supplementary Figure 1** for the funnel plot and **Supplementary Figure 2** for the forest plot).

## 4 Discussion

Previous studies have shown that thyroid dysfunction and ARC are two unrelated conditions, and their relationship has rarely been investigated. Although total thyroidectomy has been reported to cause cataracts, researchers believe that disruption of calcium and phosphorus metabolism due to removal of the parathyroid glands is the primary cause (31). Through a MR method, we found for the first time that genetically determined hypothyroidism increases the risk of developing ARC. We provided genetic support that hypothyroidism increases the risk of ARC in the European population. In sensitivity analyses, neither forest plots nor leave-one-out plots revealed SNPs that significantly influenced the results. There was no pleiotropy according to the MR-Egger intercept test and the MR-PRESSO test. In addition, the MR Steiger test indicated that there was no inverse causality with hypothyroidism and ARC.

Cataracts are the leading cause of blindness in older people, accounting for up to 51% of all cases of blindness, making them a serious global public health problem (32). Age is considered to be the most important risk factor for cataracts, which typically appear between the ages of 40 and 50, progress slowly, and typically do not affect vision until the age of 60 (2). However, age alone does not fully explain the development of ARC. Physiologically, the human lens is composed of 63% water and 35% protein (33, 34). Changes in water content and protein aggregation abnormalities in cataracts are the result of exposure to ultraviolet and x-ray radiation, steroids, and other chemicals and drugs, as well as the presence of local or systemic diseases such as diabetes, smoking, and malnutrition, rendering the normally transparent lens opaque (1, 4). Oxidative stress has also been implicated in the pathogenesis of cataract (35). Oxidative stress causes a decrease in the glutathione levels in the lens, an increase in the level of glutathione disulfide (the oxidised form of the antioxidant glutathione), and ultimately the accumulation of lens-ageing proteins, including disulfide-bound proteins (36, 37). In addition, a number of studies suggest that nutrient, vitamin and mineral intake is also associated with lens ageing (38). High protein and vitamin C intake (approximately twice the recommended levels) may delay lens ageing, and the use of various vitamin supplements and trace minerals such as selenium may be beneficial in maintaining lens health, particularly in minimizing the risk of nuclear and cortical cataracts (39, 40). These findings have implications for the prevention and treatment of cataract.

The positive causal relationship between hypothyroidism and ARC may be due to the fact that thyroid hormone deficiency affects the formation of lens proteins. In previous observational studies, patients with congenital hypothyroidism often had concomitant cataracts (41). Congenital hypothyroidism may cause thyroid hormone levels to be too low during the embryonic period, thereby affecting the normal development of ocular structures, including the lens. Alternatively, some genes may be involved in both thyroid hormone synthesis and eye development, allowing the two disorders to coexist in children with certain congenital malformations (42, 43). This is consistent with the genetic evidence we provide. In addition, THs regulate the body’s water balance, and lens water transport is a critical physiological process for maintaining lens function and image quality. The effect of THs on systemic and local water balance is important in lens lesions (34). We hypothesize that one of the reasons hypothyroidism increases the risk of ARC is due to the disruption of water metabolism associated with thyroid hormone deficiency, and that water retention may have an effect on ocular tissues and the lens.

Oxidative stress is considered to be one of the key mechanisms of cataract (35). Studies have shown that hypothyroidism increases oxidative stress, which influences the apoptosis of structural cells and inflammatory cells and alters the cytokine microenvironment balance, thereby contributing to the pathogenesis of cataract. The study by Huang et al. showed that TH can increase the lipid saturation and membrane raft lipids in human lens epithelial cell lines, thereby decreasing mitochondrial-generated reactive oxygen species and increasing the total antioxidant capacity of lens epithelial cells (44, 45). This also supports the previously held belief that mitochondrial dysfunction and metabolic dysregulation may be associated with cataracts, and that reducing the production of damaging mitochondrial ROS may ameliorate ageing eye tissue and cataract disease (46). Furthermore, THs characterise the basic metabolism of the body, particularly in relation to gastrointestinal function and visceral organs. In addition to central appetite suppression, hypothyroid patients often experience various digestive disorders, such as oesophageal dysmotility, interstitial oedema of the gastrointestinal muscular layer, and impaired liver function (47). This means that hypothyroid patients consume fewer nutrients, vitamins, and minerals than the normal population, disrupting the nutritional metabolic balance of the lens and accelerating its degeneration. Further research is needed to determine whether hypothyroidism mediates systemic diseases that contribute to the onset or progression of ARC.

However, there are some limitations to this study that need to be considered. First, all of the participants in this study were of European ancestry, so our findings may not be applicable to people of other ancestries. Second, because we used aggregated GWAS data rather than individual-level data, we were unable to stratify for factors such as age and sex to further explore the causal relationship between hypothyroidism and ARC. Next, the traits of hypothyroidism and hyperthyroidism were derived from public GWAS datasets as non-cancer disease self-report codes rather than diagnosis codes; The use of self-reported codes may lead to greater characterization of hypothyroidism and hyperthyroidism, which may increase the impact of potential heterogeneity. Fortunately, we found no significant heterogeneity through a series of quality analyses. Additionally, there are relatively few SNPs as IVs for hyperthyroidism, which may increase the risk of type II error and thus limits the ability to detect true associations between genetic factors and hyperthyroidism. Future GWAS datasets of larger, more specific diagnosis codes will improve statistical power. Finally, although MR uses genetic variation for studies that are not subject to traditional confounders such as environment and behavior, the effects of clinical interventions cannot be determined by MR analysis. Further studies are needed to explore the relationship between hypothyroidism and ARC.

## 5 Conclusion

Our study provides strong evidence that hypothyroidism is a causal determinant of ARC risk.

## Data availability statement

The original contributions presented in the study are included in the article/**Supplementary Material**, further inquiries can be directed to the corresponding authors.

## Ethics statement

Ethical review and approval were not required for this study in human subjects in accordance with the local laws and institutional requirements. Written informed consent for participation was not required for this study in accordance with national laws and institutional requirements.

## Author contributions

SL: Writing – original draft, Writing – review & editing, Data curation, Methodology, Conceptualization. QS: Writing – original draft, Writing – review & editing, Formal analysis, Software. QG: Writing – review & editing, Formal analysis. YB: Writing – review & editing, Investigation. WW: Software. XQ: Writing – original draft, Supervision, Project administration, Funding acquisition, Resources. XY: Writing – original draft, Supervision, Project administration, Funding acquisition, Resources.
