# Supplementary material for: Hypothyroidism is a causal determinant of age-related cataract risk in European population: a Mendelian randomization study
Source: Front Endocrinol (Lausanne). 2024 Feb 5;15:1254793. doi: 10.3389/fendo.2024.1254793 (PMC10874999; doi:10.3389/fendo.2024.1254793)
Supplement: Supplementary file 1 [file DataSheet_1.docx]

**TABLE S1 STROBE-MR checklist of recommended items to address in reports of Mendelian randomization studies^1 2^**

| **Item No.** | **Section** | **Checklist item** | **Page No.** | **Relevant text from manuscript** |
| --- | --- | --- | --- | --- |
| 1 | **TITLE and ABSTRACT** | Indicate Mendelian randomization (MR) as the study’s design in the title and/or the abstract if that is a main purpose of the study | 2 | Lines 28 |
|  | **INTRODUCTION** |  |  |  |
| 2 | **Background** | Explain the scientific background and rationale for the reported study. What is the exposure? Is a potential causal relationship between exposure and outcome plausible? Justify why MR is a helpful method to address the study question | 5-6 | Lines 99-122 |
| 3 | **Objectives** | State specific objectives clearly, including pre-specified causal hypotheses (if any). State that MR is a method that, under specific assumptions, intends to estimate causal effects | 5-6 | Lines 107-122 |
|  | **METHODS** |  |  |  |
| 4 | **Study design and data sources** | Present key elements of the study design early in the article. Consider including a table listing sources of data for all phases of the study. For each data source contributing to the analysis, describe the following: |  |  |
|  | a) | Setting: Describe the study design and the underlying population, if possible. Describe the setting, locations, and relevant dates, including periods of recruitment, exposure, follow-up, and data collection, when available. | 6-8  (partially) | Lines 125-160 |
|  | b) | Participants: Give the eligibility criteria, and the sources and methods of selection of participants. Report the sample size, and whether any power or sample size calculations were carried out prior to the main analysis | 6-8 | Lines 132-160 |
|  | c) | Describe measurement, quality control and selection of genetic variants | 8-9 | Lines 162-186 |
|  | d) | For each exposure, outcome, and other relevant variables, describe methods of assessment and diagnostic criteria for diseases | 6-8  (partially) | Lines 132-160 |
|  | e) | Provide details of ethics committee approval and participant informed consent, if relevant | not applicable |  |
| 5 | **Assumptions** | Explicitly state the three core IV assumptions for the main analysis (relevance, independence and exclusion restriction) as well assumptions for any additional or sensitivity analysis | 8 | Lines 162-171 |
| 6 | **Statistical methods: main analysis** | Describe statistical methods and statistics used |  |  |
|  | a) | Describe how quantitative variables were handled in the analyses (i.e., scale, units, model) | not applicable |  |
|  | b) | Describe how genetic variants were handled in the analyses and, if applicable, how their weights were selected | 9-10 | Lines 189-210 |
|  | c) | Describe the MR estimator (e.g. two-stage least squares, Wald ratio) and related statistics. Detail the included covariates and, in case of two-sample MR, whether the same covariate set was used for adjustment in the two samples | 9-10 | Lines 189-210 |
|  | d) | Explain how missing data were addressed | not applicable |  |
|  | e) | If applicable, indicate how multiple testing was addressed | not applicable |  |
| 7 | **Assessment of assumptions** | Describe any methods or prior knowledge used to assess the assumptions or justify their validity | 8 | Lines 162-171 |
| 8 | **Sensitivity analyses and additional analyses** | Describe any sensitivity analyses or additional analyses performed (e.g. comparison of effect estimates from different approaches, independent replication, bias analytic techniques, validation of instruments, simulations) | 9-10 | Lines 189-210 |
| 9 | **Software and pre-registration** |  |  |  |
|  | a) | Name statistical software and package(s), including version and settings used | 9 | Lines 203-210 |
|  | b) | State whether the study protocol and details were pre-registered (as well as when and where) | not applicable |  |
|  | **RESULTS** |  |  |  |
| 10 | **Descriptive data** |  |  |  |
|  | a) | Report the numbers of individuals at each stage of included studies and reasons for exclusion. Consider use of a flow diagram | not applicable |  |
|  | b) | Report summary statistics for phenotypic exposure(s), outcome(s), and other relevant variables (e.g. means, SDs, proportions) |  | Table 1,2 and supplementary materials |
|  | c) | If the data sources include meta-analyses of previous studies, provide the assessments of heterogeneity across these studies | not applicable |  |
|  | d) | For two-sample MR:  i.  Provide justification of the similarity of the genetic variant-exposure associations between the exposure and outcome samples  ii.  Provide information on the number of individuals who overlap between the exposure and outcome studies |  | Table 1 and table 2 |
| 11 | **Main results** |  |  |  |
|  | a) | Report the associations between genetic variant and exposure, and between genetic variant and outcome, preferably on an interpretable scale |  | Table 1 and table 2 |
|  | b) | Report MR estimates of the relationship between exposure and outcome, and the measures of uncertainty from the MR analysis, on an interpretable scale, such as odds ratio or relative risk per SD difference |  | Table 1 and table 2 |
|  | c) | If relevant, consider translating estimates of relative risk into absolute risk for a meaningful time period | 10 | Lines 238-240 |
|  | d) | Consider plots to visualize results (e.g. forest plot, scatterplot of associations between genetic variants and outcome versus between genetic variants and exposure) |  | Figure 2,3 and supplementary materials |
| 12 | **Assessment of assumptions** |  |  |  |
|  | a) | Report the assessment of the validity of the assumptions |  | Table 1 and table 2 |
|  | b) | Report any additional statistics (e.g., assessments of heterogeneity across genetic variants, such as *I^2^*, Q statistic or E-value) |  | Table 1 and table 2 |
| 13 | **Sensitivity analyses and additional analyses** |  |  |  |
|  | a) | Report any sensitivity analyses to assess the robustness of the main results to violations of the assumptions |  | Table 1 and table 2 |
|  | b) | Report results from other sensitivity analyses or additional analyses |  | Table 1 and table 2 |
|  | c) | Report any assessment of direction of causal relationship (e.g., bidirectional MR) | 10 | Lines 243-244 |
|  | d) | When relevant, report and compare with estimates from non-MR analyses | not applicable |  |
|  | e) | Consider additional plots to visualize results (e.g., leave-one-out analyses) |  | Figure 2,3 and supplementary materials |
|  | **DISCUSSION** |  |  |  |
| 14 | **Key results** | Summarize key results with reference to study objectives | 12 | Lines 275-287 |
| 15 | **Limitations** | Discuss limitations of the study, taking into account the validity of the IV assumptions, other sources of potential bias, and imprecision. Discuss both direction and magnitude of any potential bias and any efforts to address them | 15-16 | Lines 354-374 |
| 16 | **Interpretation** |  |  |  |
|  | a) | Meaning: Give a cautious overall interpretation of results in the context of their limitations and in comparison with other studies | 15-16 | Lines 354-374 |
|  | b) | Mechanism: Discuss underlying biological mechanisms that could drive a potential causal relationship between the investigated exposure and the outcome, and whether the gene-environment equivalence assumption is reasonable. Use causal language carefully, clarifying that IV estimates may provide causal effects only under certain assumptions | 12-15 | Lines 288-353 |
|  | c) | Clinical relevance: Discuss whether the results have clinical or public policy relevance, and to what extent they inform effect sizes of possible interventions | 14-15 | Lines 331-353 |
| 17 | **Generalizability** | Discuss the generalizability of the study results (a) to other populations, (b) across other exposure periods/timings, and (c) across other levels of exposure | 12-15 | Lines 275-353 |
|  | **OTHER INFORMATION** |  |  |  |
| 18 | **Funding** | Describe sources of funding and the role of funders in the present study and, if applicable, sources of funding for the databases and original study or studies on which the present study is based | 17 | Lines 400-403 |
| 19 | **Data and data sharing** | Provide the data used to perform all analyses or report where and how the data can be accessed, and reference these sources in the article. Provide the statistical code needed to reproduce the results in the article, or report whether the code is publicly accessible and if so, where | 18 | Lines 410-411 |
| 20 | **Conflicts of Interest** | All authors should declare all potential conflicts of interest | 17-18 | Lines 406-407 |

This checklist is copyrighted by the Equator Network under the Creative Commons Attribution 3.0 Unported (CC BY 3.0) license.

1. Skrivankova VW, Richmond RC, Woolf BAR, Yarmolinsky J, Davies NM, Swanson SA, et al. Strengthening the Reporting of Observational Studies in Epidemiology using Mendelian Randomization (STROBE-MR) Statement. JAMA. 2021;under review.

2. Skrivankova VW, Richmond RC, Woolf BAR, Davies NM, Swanson SA, VanderWeele TJ, et al. Strengthening the Reporting of Observational Studies in Epidemiology using Mendelian Randomisation (STROBE-MR): Explanation and Elaboration. BMJ. 2021;375:n2233.

**TABLE S2 Univariate Mendelian randomization analysis for the effects of Hypothyroidism on Age-related cataract risk.**

| **SNP** | **EAE** | **OAE** | **EAO** | **OAO** | **exposure** | | | **outcome** | | | **F** |
| --- | --- | --- | --- | --- | --- | --- | --- | --- | --- | --- | --- |
|  |  |  |  |  | **se** | **beta** | **pval** | **se** | **beta** | **pval** |  |
| rs10036386 | T | C | T | C | 0.00053396 | 0.0032141 | 1.75E-09 | 0.0117 | -0.0105 | 0.3688 | 36 |
| rs1032129 | C | A | C | A | 0.000543772 | -0.00302426 | 2.67E-08 | 0.0118 | -0.0094 | 0.4261 | 31 |
| rs10424978 | A | C | A | C | 0.00053505 | -0.0041633 | 7.21E-15 | 0.0119 | 0.0041 | 0.732501 | 61 |
| rs1045853 | C | A | C | A | 0.000553686 | -0.00337461 | 1.10E-09 | 0.0123 | -0.0169 | 0.168 | 37 |
| rs1088898 | T | G | T | G | 0.00061771 | 0.00338586 | 4.22E-08 | 0.0126 | -0.0196 | 0.1196 | 30 |
| rs10956412 | C | A | C | A | 0.000706129 | -0.00508727 | 5.84E-13 | 0.0197 | -0.0482 | 0.0143199 | 52 |
| rs11052877 | G | A | G | A | 0.000537973 | -0.00576124 | 9.31E-27 | 0.0118 | -0.0068 | 0.5658 | 115 |
| rs111618453 | A | G | A | G | 0.000583397 | 0.00577379 | 4.33E-23 | 0.0127 | -0.001 | 0.9347 | 98 |
| rs11258303 | A | C | A | C | 0.000598646 | 0.00371265 | 5.59E-10 | 0.0122 | -0.0222 | 0.0682307 | 38 |
| rs113229608 | A | C | A | C | 0.00106425 | 0.00647516 | 1.17E-09 | 0.0303 | -0.0119 | 0.6937 | 37 |
| rs11571297 | C | T | C | T | 0.000521363 | -0.00859242 | 5.34E-61 | 0.0119 | -0.0015 | 0.8978 | 272 |
| rs11675342 | T | C | T | C | 0.000525295 | 0.00461109 | 1.67E-18 | 0.0117 | 0.0126 | 0.2799 | 77 |
| rs11706511 | G | A | G | A | 0.000781286 | 0.00465932 | 2.47E-09 | 0.0166 | 0.0058 | 0.7252 | 36 |
| rs11783023 | T | C | T | C | 0.000576457 | -0.00329795 | 1.06E-08 | 0.012 | 0.0077 | 0.519501 | 33 |
| rs12117927 | A | C | A | C | 0.000532232 | 0.0032687 | 8.18E-10 | 0.0121 | 0.0196 | 0.1049 | 38 |
| rs12582330 | T | G | T | G | 0.00058512 | -0.00416475 | 1.10E-12 | 0.0124 | -0.0143 | 0.248 | 51 |
| rs12980063 | G | A | G | A | 0.000532123 | -0.00339664 | 1.74E-10 | 0.0121 | 0.0042 | 0.7257 | 41 |
| rs13090803 | T | G | T | G | 0.00063893 | 0.0050706 | 2.09E-15 | 0.0167 | 0.0015 | 0.9278 | 63 |
| rs13145888 | C | T | C | T | 0.000633817 | -0.00601133 | 2.45E-21 | 0.0176 | -0.0031 | 0.86 | 90 |
| rs13333582 | C | T | C | T | 0.00128242 | 0.00722053 | 1.80E-08 | 0.03 | 0.0039 | 0.8957 | 32 |
| rs13399762 | G | A | G | A | 0.00114529 | 0.00672799 | 4.25E-09 | 0.0308 | 0.0115 | 0.7088 | 35 |
| rs142997491 | G | A | G | A | 0.00238237 | 0.0150304 | 2.81E-10 | 0.0395 | 0.0925 | 0.0191298 | 40 |
| rs1534430 | T | C | T | C | 0.000532667 | -0.00402779 | 3.99E-14 | 0.0117 | -0.0012 | 0.9153 | 57 |
| rs1549142 | T | C | T | C | 0.000619147 | 0.0040914 | 3.90E-11 | 0.0136 | 0.0055 | 0.684301 | 44 |
| rs16903097 | G | T | G | T | 0.00076851 | -0.00452308 | 3.97E-09 | 0.0181 | 0.0229 | 0.2068 | 35 |
| rs17020110 | C | T | C | T | 0.00058753 | 0.00413049 | 2.07E-12 | 0.0138 | 0.0197 | 0.1531 | 49 |
| rs1790604 | G | A | G | A | 0.000520432 | -0.00322907 | 5.49E-10 | 0.0116 | -0.0174 | 0.1347 | 38 |
| rs2111485 | G | A | G | A | 0.000530651 | 0.00380166 | 7.84E-13 | 0.0117 | 7.00E-04 | 0.9513 | 51 |
| rs2123340 | A | G | A | G | 0.000546057 | -0.00373671 | 7.76E-12 | 0.0122 | -0.0101 | 0.4081 | 47 |
| rs221786 | C | T | C | T | 0.000819327 | 0.00494967 | 1.53E-09 | 0.0162 | 0.02 | 0.2166 | 36 |
| rs229540 | G | T | G | T | 0.000525234 | 0.00503721 | 8.83E-22 | 0.0118 | 0.0149 | 0.2063 | 92 |
| rs2412974 | T | C | T | C | 0.000540792 | -0.00311955 | 8.01E-09 | 0.0118 | -0.018 | 0.1282 | 33 |
| rs244672 | T | C | T | C | 0.000790748 | -0.00462791 | 4.84E-09 | 0.0135 | -0.0271 | 0.0454695 | 34 |
| rs2523483 | G | T | G | T | 0.00091758 | -0.00664685 | 4.37E-13 | 0.0548 | -0.0064 | 0.907 | 52 |
| rs2736191 | G | C | G | C | 0.00165851 | -0.00937316 | 1.59E-08 | 0.0257 | 0.0121 | 0.636999 | 32 |
| rs2745803 | G | A | G | A | 0.000641164 | -0.00368215 | 9.31E-09 | 0.0162 | 0.0185 | 0.2559 | 33 |
| rs28157 | T | G | T | G | 0.000558853 | -0.00349175 | 4.16E-10 | 0.0125 | 0.0109 | 0.3823 | 39 |
| rs2823272 | A | T | A | T | 0.000558859 | -0.00355403 | 2.03E-10 | 0.0134 | -0.0119 | 0.3739 | 40 |
| rs2921053 | C | G | C | G | 0.000521912 | -0.00467491 | 3.34E-19 | 0.0207 | -0.0105 | 0.611799 | 80 |
| rs3184504 | C | T | C | T | 0.000518629 | -0.0099309 | 1.10E-81 | 0.0118 | -0.0302 | 0.0102799 | 367 |
| rs3775291 | T | C | T | C | 0.000566167 | -0.00403311 | 1.05E-12 | 0.0124 | -0.0032 | 0.7956 | 51 |
| rs3850765 | C | T | C | T | 0.000526496 | 0.00313025 | 2.76E-09 | 0.0127 | 0.0404 | 0.001492 | 35 |
| rs4081335 | C | T | C | T | 0.00115775 | -0.0063816 | 3.55E-08 | 0.0234 | 0.0145 | 0.5354 | 30 |
| rs4263621 | A | G | A | G | 0.000521765 | 0.00295553 | 1.48E-08 | 0.0116 | 0.006 | 0.606599 | 32 |
| rs4276275 | T | C | T | C | 0.000519895 | 0.00340427 | 5.84E-11 | 0.0116 | -0.0068 | 0.5547 | 43 |
| rs4409785 | C | T | C | T | 0.00068747 | 0.00647971 | 4.31E-21 | 0.0155 | 0.0029 | 0.8525 | 89 |
| rs4444866 | T | C | T | C | 0.000581693 | -0.00322868 | 2.85E-08 | 0.0128 | -0.0177 | 0.1671 | 31 |
| rs60600003 | G | T | G | T | 0.000864647 | 0.00500327 | 7.19E-09 | 0.0193 | -0.0102 | 0.5972 | 33 |
| rs61759532 | T | C | T | C | 0.000618829 | 0.00419164 | 1.26E-11 | 0.0148 | 0.0045 | 0.760599 | 46 |
| rs62076510 | G | T | G | T | 0.000714773 | 0.00520033 | 3.46E-13 | 0.0154 | 0.0065 | 0.6709 | 53 |
| rs6426808 | A | G | A | G | 0.000520025 | 0.00327227 | 3.13E-10 | 0.0116 | -0.003 | 0.7954 | 40 |
| rs654537 | A | G | A | G | 0.000532298 | 0.00599281 | 2.13E-29 | 0.0116 | 0.0173 | 0.1355 | 127 |
| rs6584277 | G | A | G | A | 0.000519066 | -0.0031819 | 8.79E-10 | 0.0116 | -0.0067 | 0.5657 | 38 |
| rs66749983 | T | A | T | A | 0.000561314 | 0.00360186 | 1.39E-10 | 0.0122 | 0.015 | 0.22 | 41 |
| rs6679677 | A | C | A | C | 0.000852993 | 0.0201022 | 1.07E-122 | 0.0164 | 0.0168 | 0.3038 | 555 |
| rs6833591 | G | A | G | A | 0.000545888 | -0.00317388 | 6.10E-09 | 0.014 | -0.0233 | 0.0965006 | 34 |
| rs683763 | T | G | T | G | 0.000555044 | 0.00309064 | 2.57E-08 | 0.0118 | -0.0116 | 0.3292 | 31 |
| rs6992869 | C | T | C | T | 0.000537158 | 0.00299144 | 2.56E-08 | 0.0117 | -3.00E-04 | 0.9806 | 31 |
| rs705702 | G | A | G | A | 0.00054845 | 0.00367966 | 1.96E-11 | 0.0126 | 0.0143 | 0.2579 | 45 |
| rs7090530 | A | C | A | C | 0.000530431 | 0.00418704 | 2.94E-15 | 0.0124 | 0.0096 | 0.4374 | 62 |
| rs71508903 | T | C | T | C | 0.000661691 | 0.00625717 | 3.21E-21 | 0.015 | 0.0173 | 0.249 | 89 |
| rs736374 | A | G | A | G | 0.000540637 | 0.00412144 | 2.48E-14 | 0.0121 | 0.0021 | 0.8615 | 58 |
| rs7441808 | G | A | G | A | 0.000564386 | 0.00354074 | 3.53E-10 | 0.0128 | 0.0027 | 0.8352 | 39 |
| rs7582694 | G | C | G | C | 0.000620534 | -0.00697507 | 2.61E-29 | 0.0137 | -0.0182 | 0.1838 | 126 |
| rs761357 | T | A | T | A | 0.000537459 | 0.00331651 | 6.81E-10 | 0.0122 | 0.0234 | 0.0548795 | 38 |
| rs76428106 | C | T | C | T | 0.00238035 | 0.027135 | 4.26E-30 | 0.0539 | 0.0405 | 0.4524 | 130 |
| rs7649344 | C | T | C | T | 0.000521217 | -0.00296836 | 1.23E-08 | 0.012 | 2.00E-04 | 0.9892 | 32 |
| rs76518703 | G | A | G | A | 0.00122104 | -0.0100896 | 1.42E-16 | 0.031 | 0.0182 | 0.5586 | 68 |
| rs7768019 | G | C | G | C | 0.000601065 | -0.00812346 | 1.31E-41 | 0.013 | 0.0373 | 0.0041830 | 183 |
| rs7905731 | C | T | C | T | 0.000528315 | -0.00314417 | 2.66E-09 | 0.012 | 0.0119 | 0.3248 | 35 |
| rs8008961 | T | C | T | C | 0.000577942 | -0.00336955 | 5.54E-09 | 0.0126 | -0.0149 | 0.2382 | 34 |
| rs8043085 | T | G | T | G | 0.000614119 | 0.00425081 | 4.47E-12 | 0.0138 | 0.0113 | 0.4139 | 48 |
| rs8054578 | G | A | G | A | 0.000623096 | -0.00358256 | 8.95E-09 | 0.0153 | 0.02 | 0.1911 | 33 |
| rs853303 | G | A | G | A | 0.000534335 | -0.00374837 | 2.30E-12 | 0.0125 | -0.0065 | 0.6055 | 49 |
| rs911760 | A | C | A | C | 0.000672507 | 0.00418051 | 5.10E-10 | 0.0142 | 0.0138 | 0.3322 | 39 |
| rs925489 | T | C | T | C | 0.000550975 | 0.00985707 | 1.52E-71 | 0.0121 | -0.0193 | 0.1109 | 320 |
| rs926103 | C | T | C | T | 0.000547963 | -0.00298868 | 4.92E-08 | 0.0119 | -0.0067 | 0.5777 | 30 |
| rs9277569 | T | C | T | C | 0.000839758 | 0.00840785 | 1.36E-23 | 0.0165 | -0.0183 | 0.2667 | 100 |
| rs933243 | A | C | A | C | 0.000549604 | -0.00557022 | 3.90E-24 | 0.012 | 0.0017 | 0.8904 | 103 |
| rs9497965 | T | C | T | C | 0.000528511 | 0.00364705 | 5.19E-12 | 0.0122 | 0.0207 | 0.0906505 | 48 |
| rs9815073 | A | C | A | C | 0.000567968 | -0.00705726 | 1.94E-35 | 0.0125 | 4.00E-04 | 0.9728 | 154 |

SNP: single nucleotide polymorphism; EAE: effect allele exposure; OAE: other allele exposure; EAO: effect allele outcome; OAO: other allele outcome; SE: standard error; F:F value;

**TABLE S3 Univariate Mendelian randomization analysis for the effects of Hyperthyroidism on Age-related cataract risk.**

| **SNP** | **EAE** | **OAE** | **EAO** | **OAO** | **exposure** | | | **outcome** | | | **F** |
| --- | --- | --- | --- | --- | --- | --- | --- | --- | --- | --- | --- |
|  |  |  |  |  | **se** | **beta** | **pval** | **se** | **beta** | **pval** |  |
| rs2160215 | C | T | C | T | 0.000218 | 0.002516 | 8.90E-31 | 0.015366 | -0.02367 | 0.123412 | 133 |
| rs28360997 | A | G | A | G | 0.000296 | -0.00172 | 6.01E-09 | 0.021445 | 0.013463 | 0.530161 | 34 |
| rs3087243 | A | G | A | G | 0.000212 | -0.00200 | 3.80E-21 | 0.014861 | -0.02519 | 0.090016 | 89 |
| rs6679677 | A | C | A | C | 0.000347 | 0.002618 | 4.42E-14 | 0.019872 | 0.003985 | 0.841047 | 57 |

SNP: single nucleotide polymorphism; EAE: effect allele exposure; OAE: other allele exposure; EAO: effect allele outcome; OAO: other allele outcome; SE: standard error; F: F value;

**TABLE S4 Univariate Mendelian randomization analysis for the effects of FT4 on Age-related cataract risk.**

| SNP | EAE | OAE | EAO | OAO | exposure | | | outcome | | | F |
| --- | --- | --- | --- | --- | --- | --- | --- | --- | --- | --- | --- |
|  |  |  |  |  | se | beta | pval | se | beta | pval |  |
| rs10119187 | T | C | T | C | 0.009 | 0.05 | 4.10E-09 | 0.0165 | -0.0388 | 0.0183202 | 31 |
| rs10739496 | T | C | T | C | 0.007 | 0.078 | 4.20E-30 | 0.0121 | -0.0212 | 0.0808593 | 124 |
| rs10818937 | T | C | T | C | 0.007 | -0.048 | 1.30E-11 | 0.0123 | -0.0128 | 0.2983 | 47 |
| rs10946313 | T | C | T | C | 0.007 | 0.046 | 2.30E-11 | 0.0117 | 0.0063 | 0.5898 | 43 |
| rs11039355 | T | C | T | C | 0.007 | -0.039 | 3.50E-08 | 0.0125 | -0.0058 | 0.6426 | 31 |
| rs113107469 | T | C | T | C | 0.022 | 0.2 | 1.00E-19 | 0.0413 | 0.0399 | 0.3346 | 83 |
| rs12907106 | C | G | C | G | 0.007 | -0.041 | 3.70E-08 | 0.0122 | 1.00E-04 | 0.9919 | 34 |
| rs17185536 | T | C | T | C | 0.008 | 0.073 | 1.90E-19 | 0.0144 | 0.0318 | 0.0273401 | 83 |
| rs2235544 | A | C | A | C | 0.007 | 0.139 | 4.20E-101 | 0.0117 | -0.0027 | 0.8204 | 394 |
| rs225014 | T | C | T | C | 0.007 | 0.054 | 1.80E-15 | 0.0129 | 0.0029 | 0.8201 | 60 |
| rs4149056 | T | C | T | C | 0.009 | -0.051 | 1.30E-08 | 0.0145 | 0.0407 | 0.0048859 | 32 |
| rs4842131 | T | C | T | C | 0.008 | -0.104 | 7.70E-44 | 0.0116 | -0.0021 | 0.8545 | 169 |
| rs4954192 | T | C | T | C | 0.007 | -0.041 | 8.40E-09 | 0.0118 | 0.0044 | 0.7099 | 34 |
| rs56069042 | A | G | A | G | 0.019 | 0.106 | 1.20E-08 | 0.0289 | -0.0646 | 0.02556 | 31 |
| rs67583169 | C | G | C | G | 0.01 | 0.061 | 1.00E-10 | 0.014 | 0.0117 | 0.4028 | 37 |
| rs6785807 | A | G | A | G | 0.009 | -0.059 | 2.50E-10 | 0.0132 | 0.0031 | 0.8129 | 43 |
| rs6854291 | A | G | A | G | 0.011 | 0.117 | 1.30E-24 | 0.0254 | 0.0254 | 0.3171 | 113 |
| rs8063103 | C | G | C | G | 0.009 | -0.052 | 1.60E-08 | 0.0187 | 0.0273 | 0.1449 | 33 |
| rs9356988 | A | G | A | G | 0.007 | -0.051 | 3.60E-12 | 0.0121 | 0.009 | 0.4557 | 53 |
| rs10119187 | T | C | T | C | 0.009 | 0.05 | 4.10E-09 | 0.0165 | -0.0388 | 0.0183202 | 31 |
| rs10739496 | T | C | T | C | 0.007 | 0.078 | 4.20E-30 | 0.0121 | -0.0212 | 0.0808593 | 124 |
| rs10818937 | T | C | T | C | 0.007 | -0.048 | 1.30E-11 | 0.0123 | -0.0128 | 0.2983 | 47 |
| rs10946313 | T | C | T | C | 0.007 | 0.046 | 2.30E-11 | 0.0117 | 0.0063 | 0.5898 | 43 |

SNP: single nucleotide polymorphism; EAE: effect allele exposure; OAE: other allele exposure; EAO: effect allele outcome; OAO: other allele outcome; SE: standard error; F:F value;

**TABLE S5 Univariate Mendelian randomization analysis for the effects of TSH on Age-related cataract risk.**

| **SNP** | **EAE** | **OAE** | **EAO** | **OAO** | **exposure** | | | **outcome** | | | **F** |
| --- | --- | --- | --- | --- | --- | --- | --- | --- | --- | --- | --- |
|  |  |  |  |  | **se** | **beta** | **pval** | **se** | **beta** | **pval** |  |
| rs1042673 | A | G | A | G | 0.006 | -0.055 | 3.60E-19 | 0.0117 | 0.0051 | 0.662599 | 84 |
| rs1045476 | A | G | A | G | 0.008 | 0.049 | 2.40E-09 | 0.0148 | 0.0089 | 0.5495 | 38 |
| rs1079418 | A | G | A | G | 0.007 | 0.101 | 8.20E-53 | 0.0131 | 0.0085 | 0.5142 | 208 |
| rs10814915 | T | C | T | C | 0.006 | 0.042 | 5.10E-12 | 0.0116 | -0.0016 | 0.891 | 49 |
| rs10917469 | A | G | A | G | 0.009 | 0.111 | 4.00E-39 | 0.0177 | -0.0149 | 0.4007 | 152 |
| rs10957494 | A | G | A | G | 0.007 | -0.04 | 1.10E-09 | 0.0125 | 0.008 | 0.5248 | 33 |
| rs11255790 | T | C | T | C | 0.007 | -0.041 | 6.80E-10 | 0.0148 | 0.0241 | 0.1047 | 34 |
| rs1157994 | A | G | A | G | 0.016 | -0.09 | 5.30E-09 | 0.0288 | 0.0153 | 0.596 | 32 |
| rs11732089 | T | C | T | C | 0.008 | 0.115 | 1.70E-51 | 0.0177 | 0.0022 | 0.8998 | 207 |
| rs1203944 | T | C | T | C | 0.007 | -0.051 | 2.40E-12 | 0.0143 | -7.00E-04 | 0.961 | 53 |
| rs12284404 | A | G | A | G | 0.007 | -0.067 | 2.50E-22 | 0.0127 | 0.0182 | 0.1516 | 92 |
| rs12390237 | A | G | A | G | 0.007 | -0.046 | 1.70E-11 | 0.01 | -0.0045 | 0.649999 | 43 |
| rs1265091 | T | C | T | C | 0.009 | 0.057 | 3.20E-11 | 0.0174 | -0.0051 | 0.767701 | 40 |
| rs12893151 | A | C | A | C | 0.008 | -0.062 | 1.00E-15 | 0.0133 | -0.008 | 0.5457 | 60 |
| rs13015993 | A | G | A | G | 0.007 | 0.082 | 4.50E-32 | 0.012 | -0.038 | 0.0015140 | 137 |
| rs13100823 | T | C | T | C | 0.007 | -0.041 | 6.80E-10 | 0.0125 | 0.0196 | 0.117 | 34 |
| rs13329353 | T | C | T | C | 0.007 | 0.061 | 5.20E-21 | 0.0122 | 0.0041 | 0.7392 | 76 |
| rs1663070 | T | C | T | C | 0.007 | -0.046 | 3.50E-11 | 0.0138 | -0.0034 | 0.8046 | 43 |
| rs17020122 | T | C | T | C | 0.011 | 0.104 | 5.30E-20 | 0.0181 | 0.0026 | 0.8842 | 89 |
| rs17477923 | T | C | T | C | 0.007 | 0.083 | 2.60E-33 | 0.0125 | -0.0233 | 0.0628001 | 141 |
| rs17767491 | A | G | A | G | 0.007 | 0.088 | 3.40E-42 | 0.0125 | -0.0195 | 0.1209 | 158 |
| rs2127387 | A | G | A | G | 0.006 | 0.144 | 1.10E-117 | 0.0117 | -0.0117 | 0.3194 | 576 |
| rs2439301 | A | G | A | G | 0.008 | -0.059 | 8.20E-15 | 0.0131 | 0.0217 | 0.0963696 | 54 |
| rs28502438 | T | C | T | C | 0.006 | 0.034 | 3.70E-08 | 0.0121 | 0.0045 | 0.7089 | 32 |
| rs30227 | T | C | T | C | 0.006 | -0.047 | 7.60E-14 | 0.012 | 0.018 | 0.1339 | 61 |
| rs334725 | A | G | A | G | 0.015 | 0.174 | 2.40E-32 | 0.0414 | 0.0282 | 0.4963 | 135 |
| rs398745 | A | C | A | C | 0.006 | -0.052 | 4.00E-17 | 0.0119 | -0.0069 | 0.5629 | 75 |
| rs4445669 | T | C | T | C | 0.006 | -0.04 | 5.80E-11 | 0.0116 | -0.0093 | 0.4234 | 44 |
| rs4804413 | T | C | T | C | 0.006 | 0.053 | 8.60E-18 | 0.0118 | 0.0099 | 0.4038 | 78 |
| rs4933466 | A | G | A | G | 0.006 | 0.04 | 5.10E-10 | 0.0118 | 0.0131 | 0.2679 | 44 |
| rs56009477 | A | G | A | G | 0.008 | 0.052 | 3.70E-10 | 0.0173 | 0.0199 | 0.2501 | 42 |
| rs59381142 | A | G | A | G | 0.008 | -0.058 | 1.70E-14 | 0.015 | 6.00E-04 | 0.97 | 53 |
| rs6724073 | T | C | T | C | 0.008 | 0.051 | 1.30E-10 | 0.012 | 0.0098 | 0.4112 | 41 |
| rs7329958 | T | C | T | C | 0.007 | -0.044 | 1.10E-11 | 0.0122 | 0.0077 | 0.5241 | 40 |
| rs744103 | A | T | A | T | 0.007 | 0.092 | 6.70E-41 | 0.0125 | 0.0166 | 0.183 | 173 |
| rs8015085 | A | G | A | G | 0.008 | 0.067 | 2.40E-18 | 0.0151 | -0.0093 | 0.538801 | 70 |
| rs9298749 | A | C | A | C | 0.006 | -0.039 | 8.80E-10 | 0.0117 | 0.0016 | 0.8924 | 42 |
| rs9497965 | T | C | T | C | 0.006 | 0.044 | 9.80E-13 | 0.0122 | 0.0207 | 0.0906505 | 54 |

SNP: single nucleotide polymorphism; EAE: effect allele exposure; OAE: other allele exposure; EAO: effect allele outcome; OAO: other allele outcome; SE: standard error; F: F value.


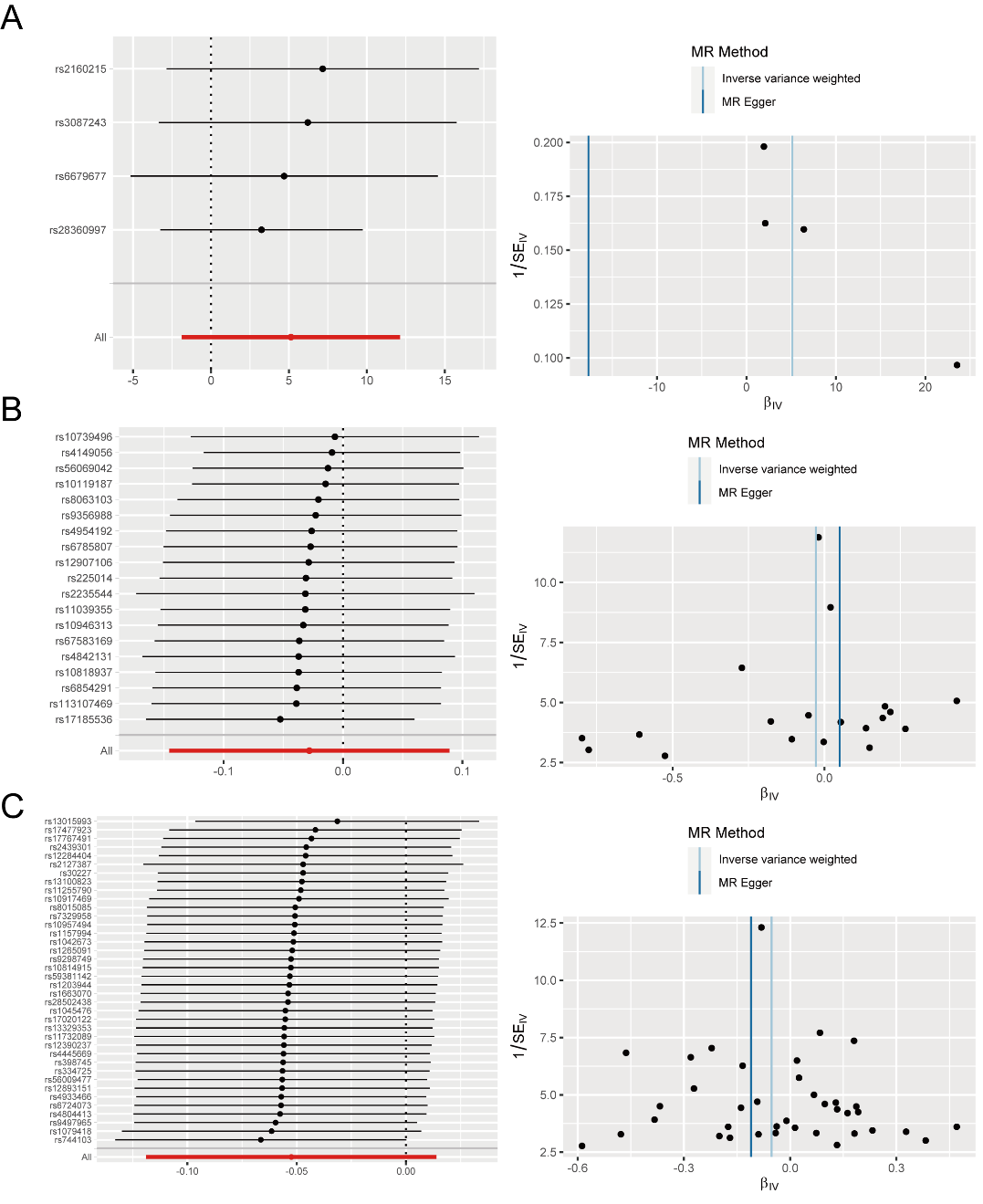


**Figure S1** **Forest plot for leave-one-out analysis and** **funnle plots and for Mendelian randomization (MR) analyses of the Causal relationship between the remaining three types of thyroid dysfunction and age-related cataract. (A) Hyperthyroidism-ARC. (B).FT4-ARC. (c).TSH-ARC**


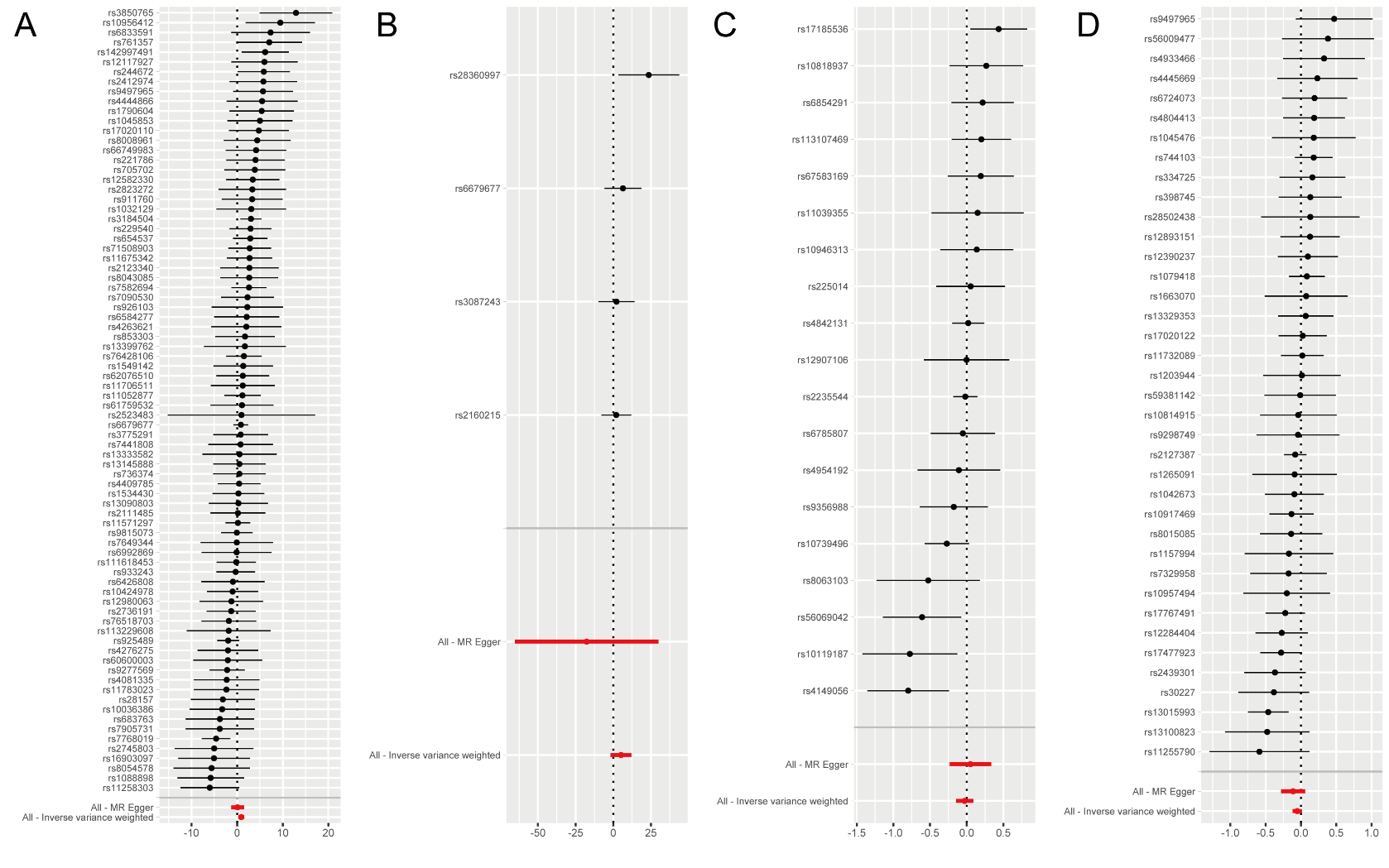


**Figure S2** **Forest plots for Mendelian randomization (MR) analyses of the Causal relationship between thyroid dysfunction and age-related cataract.(A) Hypothyroidism-ARC.(B)Hyperthyroidism- ARC.(C)FT4- ARC.(D)TSH- ARC**
